# Health advocates to address social adversity in pediatric liver transplantation: A clinical trial

**DOI:** 10.1097/HC9.0000000000000999

**Published:** 2026-07-15

**Authors:** Sharad I. Wadhwani, Bethany Reyna, Andrea Huerta, John C. Bucuvalas, Laura M. Gottlieb, Catherine Lee, Courtney R. Lyles, Sue J. Rhee, Amy M. Shui, Jennifer C. Lai

**Affiliations:** 1 Department of Pediatrics, University of California San Francisco, San Francisco, California, USA; 2 Department of Pediatrics, Icahn School of Medicine at Mount Sinai, New York, New York, USA; 3 Department of Family & Community Medicine, University of California San Francisco, California, USA; 4 Department of Medicine, University of California San Francisco, San Francisco, California, USA; 5 Center for Healthcare Policy & Research, University of California Davis, Davis, California, USA

**Keywords:** intervention research, liver transplant outcomes, pediatrics, social adversity, social determinants of heath

## Abstract

**Background:**

Children undergoing liver transplantation experience socioeconomic disparities in long-term outcomes, partly due to unaddressed material economic hardships such as food insecurity and housing instability. Health Advocates—trained lay health workers who assist families in navigating social and medical systems—may mitigate these disparities. We pilot-tested a Health Advocate intervention (NCT05700799) to assess feasibility, acceptability, and preliminary effects on caregiver experience and clinical outcomes in pediatric liver transplant recipients.

**Methods:**

In this single-center study, 18 caregivers/child dyads participated in a 90-day Health Advocate program; they were provided weekly support from trained navigators. Navigators helped to identify and connect participants with community-based resources and communicated reported social risks to the transplant team.

**Results:**

The patients’ median age at transplant was 1.1 (0.7, 4.6) and the median age at study enrollment was 8.6 (4.3, 13.1) years; 83% remained in the intervention until their needs were met or the end of the 12-week intervention. Caregivers reported improvement in comfort discussing social risks with the transplant team and improved overall care experience (both median 1-point change). Clinical parameters, including the Medication Level Variability Index, trended toward improvements. Qualitative analysis demonstrated high enthusiasm for the intervention, with themes of enhanced communication, reduced stress, and practical assistance addressing diverse social needs.

**Conclusions:**

A Health Advocate intervention for pediatric liver transplant families was feasible, acceptable, and associated with improved caregiver experiences. These preliminary findings support testing this intervention in a multi-center effectiveness and implementation trial to evaluate its impact on improving longer-term transplant outcomes.

## Graphical abstract

**Figure:**
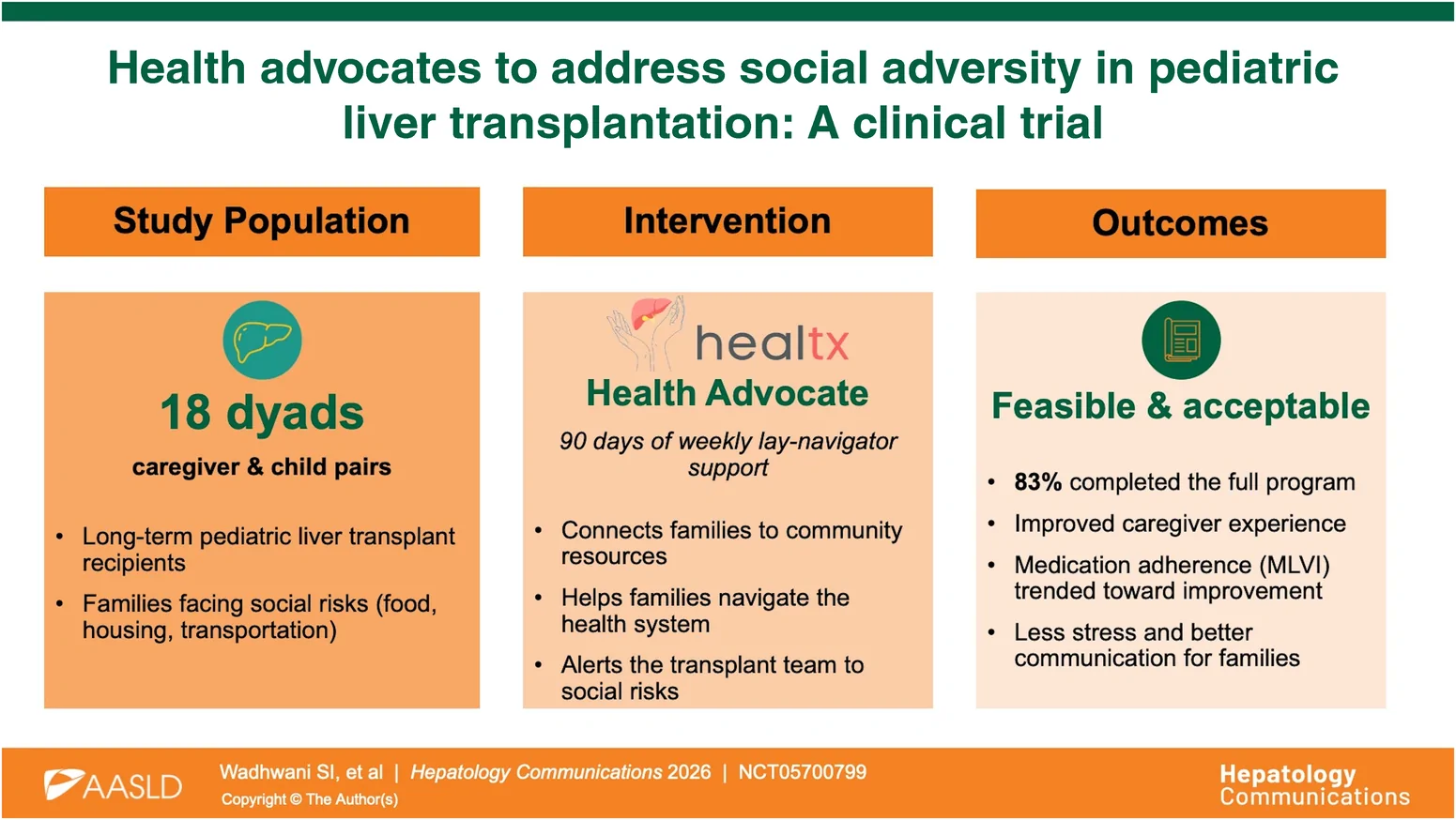

## INTRODUCTION

Pediatric liver transplantation is the only life-saving intervention for children with end-stage liver disease. While there have been tremendous surgical advances over the past 50 years, long-term outcomes remain sub-optimal.^1^^,^^2^ Moreover, racial and socioeconomic disparities pervade the transplant continuum, starting pre-transplant and extending through long-term survival.^3^^–^^6^ These disparities may be explained, in part, by the added material economic hardships experienced by families with limited financial means. In the previous work, we have found that households experiencing material economic hardship (eg, food insecurity, housing instability, transportation challenges, or utility needs) are at increased risk of T-cell-mediated rejection within the first year following liver transplantation.^7^^,^^8^ In addition, we have identified several aspects of transplant care that exacerbate economic hardship—for example, the compounded stress of lost wages, increased out-of-pocket expenses, and increased caregiving responsibilities—and others that affect the ability of the healthcare teams to address those stressors, including variable social worker availability and engagement.^9^^,^^10^

Health Advocates, or lay healthcare members with no formal medical training, have been used in other healthcare settings as a strategy to address healthcare disparities.^11^^,^^12^ These team members can help families identify and connect with community-based resources, navigate their medical care, and alert the medical team to specific hardships that the family may be experiencing. In pediatric primary care, for example, this model has resulted in improved health outcomes and decreased healthcare utilization.^11^^–^^13^ To adapt this program for pediatric liver transplant recipients, we conducted in-depth qualitative interviews with patient caregivers and transplant team members to identify gaps in the current care paradigm, and then we engaged them in a set of human-centered design focus groups to develop the intervention components of a pediatric liver transplant-specific health advocate intervention (Figure 1).

**FIGURE 1:**
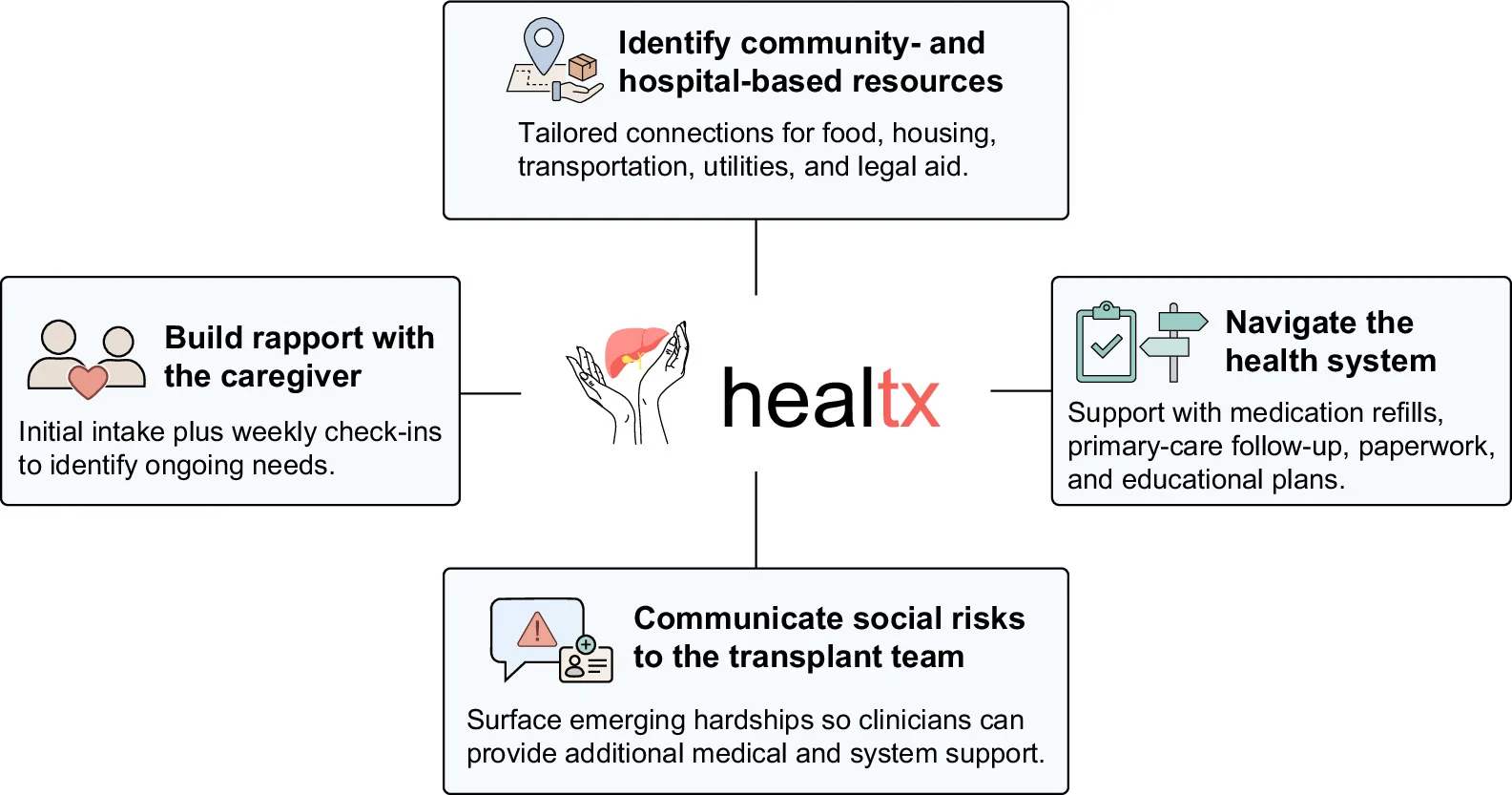
Conceptual model of the Health Advocate role. The conceptual model highlights how the Health Advocate supports caregivers and transplant teams.

In this study, we piloted the new program to assess the feasibility and acceptability of the refined Health Advocate program to support children and families after liver transplant (*HEAL-Tx Pilot, NCT:* 05700799). We organized the program evaluation around the Reach, Effectiveness, Adoption, Implementation, Maintenance (RE-AIM) framework, an implementation science framework for evaluating especially suitable for evaluating complex health system interventions.^14^

## METHODS

This study was conducted in accordance with both the Declarations of Helsinki and Istanbul; this study was reviewed by the University of California, San Francisco IRB (22-36504) and all participants provided written informed consent. This was a single-center pilot study at the University of California San Francisco (UCSF). The clinicians on the pediatric liver transplant team at UCSF include 6 hepatologists, a surgical director, a nurse practitioner, a nurse coordinator, and a social worker. Neither the transplant program nor the children’s hospital has any existing navigation programs (eg, a cultural navigator program).

### Health Advocate intervention

#### Health Advocate model adaptation

We engaged transplant stakeholders (ie, clinicians and caregivers) in a set of human-centered design focus groups to adapt an established Health Advocate program to meet the needs of transplant families.^15^ The original Health Advocate intervention was developed by Laura M. Gottlieb and is currently implemented at a large safety-net pediatric primary care practice. Key adaptations included: (1) helping families identify both community-based and hospital-based resources, and (2) alerting the transplant team to the social risks families are experiencing.

#### Health Advocate training

Prior to enrolling participants, our Health Advocate (Bethany Reyna, a clinical research coordinator) underwent training with the supervisor of the established Health Advocate program to learn how the Health Advocates support families in identifying and connecting with community-based resources. This training included a 1-hour one-on-one session with the supervisor, a 2-hour overview of the established Health Advocate program, workflow, and resource navigation tools, a 1-hour shadowing session with an established Health Advocate, and a 1-hour meeting with a social work manager to review escalation procedures, reportable events, and approaches to navigating patient situations.

#### Health Advocate intervention overview

The pilot Health Advocate intervention lasted 90 days and focused on (1) building rapport with the caregiver, (2) identifying and connecting families with community-based resources to address existing hardships, and (3) alerting the transplant team to new hardships the family was encountering. At the start of the intervention, the Health Advocate conducted an intake interview to understand each family’s social risks and assets. Then, the Health Advocate used existing information about community resources to provide tailored support based on identified risks. Examples included finding neighborhood food pantries, helping families complete Supplemental Nutrition Assistance Program (SNAP) applications, and finding free legal aid. After the initial consultation, the Health Advocate called families weekly to identify ongoing needs and help families address those needs. The Health Advocate would also communicate updates to the clinical team on the families’ needs so they could provide additional support as necessary (eg, writing a letter of medical necessity).

### Participants

We intentionally kept our inclusion criteria broad because we wanted to maximize enrollment rate to refine the intervention and identify implementation challenges. Therefore, we did not specifically select patients on the basis of any specific social risk factor. Rather, we enrolled patients based on a holistic assessment of benefit. Weekly, we reviewed the upcoming patients seen in the outpatient clinic for liver transplant follow-up. We reviewed the results of the clinic-based Accountable Healthcare Communities 10-question social risk screening. We approached families with reported social risks (ie, food insecurity, housing instability, transportation challenges, and/or utility resource needs) or those whom the transplant team believed could be a good fit. Inclusion criteria included: (1) pediatric liver transplant recipients <18 years of age at the time of enrollment, (2) parent/legal guardian able to provide informed consent and willing to participate, and (3) transplant team identified as having specific hardships that the Health Advocate could support. Exclusion criteria included: (1) the child being a ward of the state, (2) the family being non-English/non-Spanish speaking, (3) non-US residents, and (4) the liver recipient being older than 18 years at the time of enrollment.

### Data collection procedures

We collected qualitative and quantitative data during the intervention. At the start of the intervention, caregivers completed a survey and an in-depth interview, which both helped the Health Advocate understand their social risks and assets and served as baseline research data. Caregiver-reported survey measures included select questions on patient experience (from the National Survey for Children’s Health), experience with social care, social risk assessment (using the Accountable Healthcare Communities tool and single financial strain question), and the global assessment of child health question. The research team conducted in-depth interviews at 30, 60, and 90 days during the intervention. A clinical research coordinator (CRC) (Andrea Huerta), who was not serving as a Health Advocate, conducted all in-depth interviews to encourage honest dialogue. Participant caregivers completed a post-intervention survey at the conclusion of the intervention. EHR data from the pediatric patient were extracted 6 months after enrollment in the study.

### Study measures

#### Intervention intensity and fidelity

We kept detailed journal entries each time the Health Advocate contacted a participant, a community-based resource, or a transplant team member on behalf of a particular participant. The Health Advocate also documented the purpose of each contact.

#### Primary outcome

The primary outcome was changes in caregiver experiences with social risk discussions. We asked about their overall experience discussing their social risks with the transplant team on a 5-point Likert scale. We also summed questions (each with a 5-point Likert scale response) on (1) overall experience, (2) how comfortable parent felt, (3) how respected the parent felt, and (4) whether it was a good use of time to discuss social and economic issues, where the minimum score was 4 and the maximum score was 20, and higher values indicated more favorable experiences. These questions were derived from qualitative data on factors driving patient experiences of social care.^16^

#### Secondary outcomes

1. Sensitivity to the caregivers’ needs. We used a set of 5 questions focused on sensitivity to the caregiver's needs, each rated on a 4-point Likert scale. “During the past 12 months, how often did this child’s doctors or other healthcare providers: (1) spend enough time with this child?, (2) listen carefully to you?, (3) show sensitivity to your family’s values and customs?, (4) provide the specific information you needed concerning this child?, (5) help you feel like a partner in this child’s care?” We calculated the composite score, where the minimum score was 5, and the maximum score was 20, and lower values indicated more favorable care experiences. These questions were taken from the National Survey of Children’s Health.^17^
2. Shared decision-making. A set of 3 questions focused on shared decision-making; responses to each question were rated on a 4-point Likert scale. “How often did this child’s doctors or other healthcare providers: (1) discuss with you the range of options to consider for their healthcare or treatment?, (2) make it easy for you to raise concerns or disagree with recommendations for this child’s health care?, (3) work with you to decide together which health care and treatment choices would be best for this child?” We calculated the composite score, where the minimum score was 3, and the maximum score was 12, and lower values indicated more favorable care experiences. These questions were taken from the National Survey of Children’s Health.^17^
3. Caregiver-perceived global assessment of child health. We asked a single question, assessed on a 5-point Likert scale, on caregivers’ perceptions of their child’s health.^11^

#### Blood biomarker changes

We measured objective laboratory measures, including changes in the Medication Level Variability Index (MLVI) and changes in alanine aminotransferase (ALT) and gamma-glutamyl transferase (GGT).

#### Qualitative interviews

We collected qualitative assessments of intervention effectiveness and specific Health Advocate functions most helpful to caregivers and transplant team members based on interviews with caregiver participants throughout the intervention.

### Analysis plan

The primary aim of this pilot study was to pilot-test the feasibility and acceptability of the Health Advocate intervention. Since this pilot study was not intended to test the hypothesis that the intervention improved parent-reported or patient health outcomes, nor provide effect size estimates, we did not report *p*-values.^18^^,^^19^ For survey data, we developed Sankey diagrams to show changes in response from pre-intervention to post-intervention.

Qualitative interviews were audio recorded and transcribed (or translated and transcribed for Spanish-speaking families) using a professional transcription service (TranscriptionWing). We structured a qualitative assessment around RE-AIM, an implementation science framework. First, we generated a codebook with example quotes for each construct. The primary qualitative codes from this framework were “effectiveness”, “adoption”, and “implementation”. Two clinical research coordinators (Bethany Reyna and Andrea Huerta) coded all interview transcripts and Health Advocate journey entries. After they each separately coded a transcript, they would meet to discuss the codes and adjudicate any disagreements. After all transcripts were coded, we thematically analyzed each code to identify themes. We met as a group to discuss emerging themes. All qualitative data were analyzed using Dedoose.

This study was approved by the UCSF IRB (22-36504), and all caregiver participants provided informed consent.

## RESULTS

We recruited N=18 caregiver/patient dyads (Table 1). The patients were a median of 1.1 years at the time of their liver transplant, and a median of 8.6 years at the time of enrollment in this pilot study; therefore, participants in this intervention were long-term pediatric liver transplant recipients. Most of the patients were female, of “other” race, and of Hispanic ethnicity. The median ALT and GGT at time of enrollment were 25 (IQR 19, 42) and 34 (IQR 16, 44), respectively. Of the 18 caregiver/patient dyads that consented to enrollment, 15 (83%) stayed in the intervention for the entire 12-week duration. Those who left the study early withdrew before intervention activities commenced (N=2) or were lost to follow-up after 11 weeks of intervention (N=1). Supplemental Figure S1, http://links.lww.com/HC9/C402, depicts a consort diagram (Table 1).

**TABLE 1:** Demographic and clinical characteristics of N=18 patients enrolled in the study

Variable	Median [IQR] or N (%)
Patient age at transplant, years	1.1 [0.7, 4.6]
Patient age at study enrollment, years	8.6 [4.3, 13.1]
Female	13 (81)
Patient race	
White	2 (13)
Black	1 (6)
Asian or Pacific Islander	1 (6)
Other	12 (75)
Ethnicity	
Hispanic or Latino	15 (94)
Not Hispanic or Latino	1 (6)
Transplant indication	
Biliary atresia	8 (44)
Acute liver failure	2 (11)
Metabolic/genetic	3 (17)
Liver tumor	2 (11)
Other/unknown	3 (17)

### Intervention intensity and fidelity

The Health Advocate made a median of 12 (IQR 10, 18) calls to each caregiver. The Health Advocate made a median of 13 (IQR 6, 17) resource referrals per participant, and 94% of caregivers established a connection with the resource. The Health Advocate alerted the clinical team to concerns she identified for 81% of participants, and the clinical team adjusted care plans based on that information for 44% of the participants.

### Changes in experience discussing social risks

For overall experience, we observed improvements, with before and after median scores of 4 and 5, respectively. For the summed responses to (1) overall experience, (2) how comfortable the parent felt, (3) how respected the parent felt, and (4) whether it was a good use of time to discuss social and economic issues, we found an improvement from before (16.5; IQR 13, 18) and after (17.5; IQR 14, 20) the intervention, respectively.

### Changes in caregiver experience

#### Sensitivity to caregiver needs

The median composite score decreased from 8 (IQR 5, 11) pre-intervention to 6 (IQR 5, 8) post-intervention, indicating improvement in caregiver experience.

#### Sharad decision-making

Before and after intervention, the median composite scores were 4 (IQR 3, 6) and 3 (IQR 3, 4), respectively, with lower scores reflecting more positive experiences.

#### Overall perceptions of child health

The median pre-intervention and post-intervention caregiver perception of overall child health was 3 (IQR 2, 3) and 2 (IQR 1, 3), respectively, with lower scores indicating better perceived health.

Figure 2 depicts Sankey diagrams for all parent-reported outcome measures.

**FIGURE 2:**
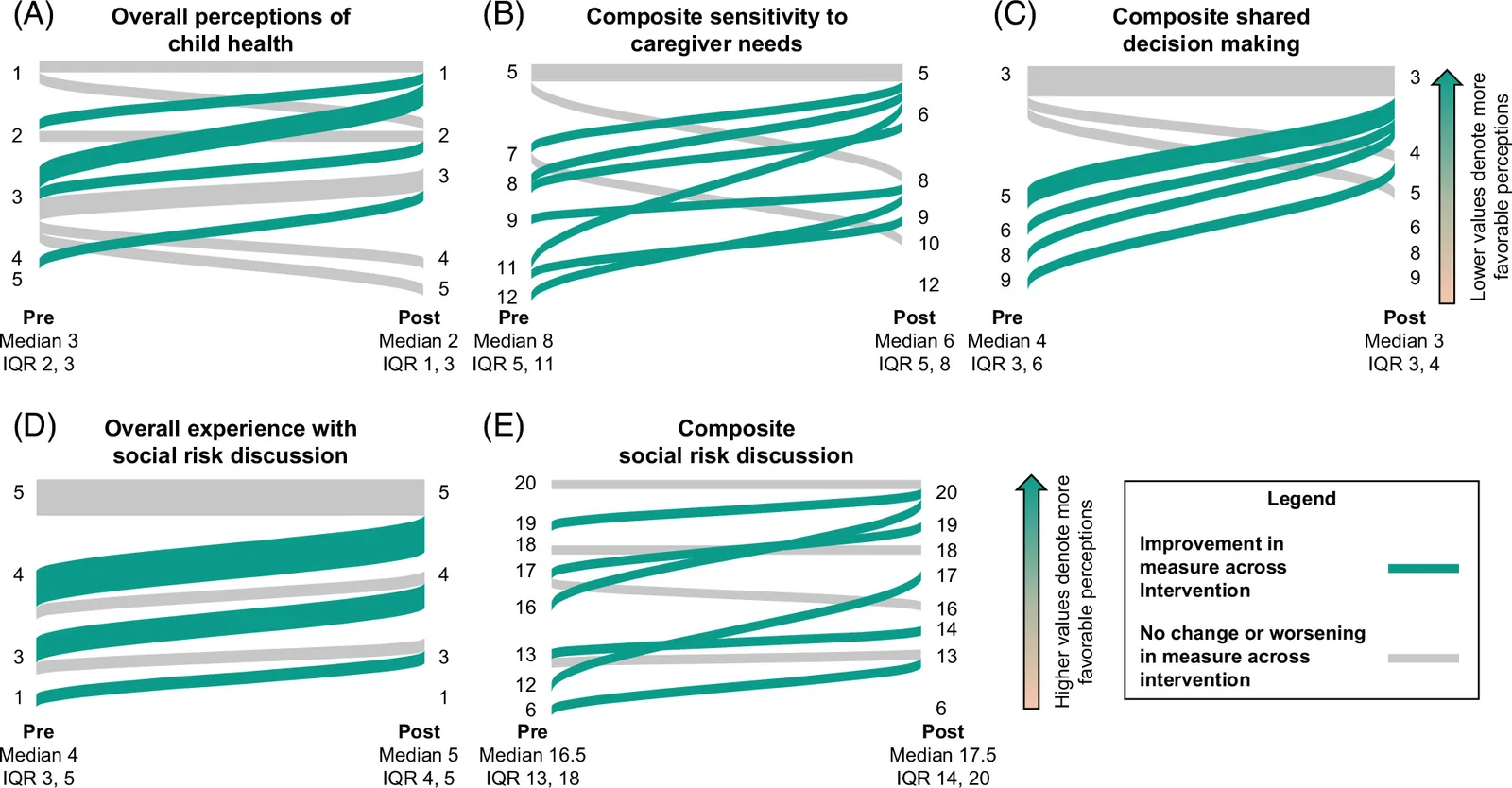
Sankey diagrams for pre-intervention and post-intervention parent-reported outcomes. Green lines depict participant-level improvements in reported caregiver experiences pre-intervention to post-intervention. Gray lines depict participant-level worsening or constant values pre-intervention to post-intervention. Axes have been oriented to show improved values at the top.

### Changes to MLVI and other clinical parameters

The median MLVI improved from the time of enrollment to the end of intervention and at 6 months after enrollment; MLVI values at these time points were 1.2 (IQR 0.6, 2.1), 1.0 (0.6, 1.5), and 0.8 (IQR 0.5, 2.0), respectively. The median ALT at time of enrollment and at 6 months after enrollment were 25 (IQR 19, 42) and 24 (IQR 20, 40), respectively. The median GGT at time of enrollment and at 6 months after enrollment were 34 (IQR 16, 44) and 24 (IQR 12, 53), respectively.

### Qualitative insights around intervention delivery

Overall, caregivers reported high levels of enthusiasm for the intervention. They noted 3 key Health Advocate characteristics that contributed to their favorable impressions: (1) helped families communicate with the medical team; (2) helped families to address multiple, often disparate, needs; (3) were proactive and accessible. These characteristics together contributed to caregivers’ reports of reduced levels of stress and burden (Table 2).

1. *Improved communication with the medical team.* Participants described that the Health Advocate enabled improved communication with the medical team. They reported feeling better supported by the medical team because the Health Advocate was able to help them better communicate with their clinicians.
  “Yes, because sometimes I had questions for the doctors, and she made it easier for me to communicate with them.”—ID 40
2. *Addressed multiple disparate tasks.* Participants highlighted how the Health Advocate helps families address multiple, disparate tasks. Some participants appreciated help with medication refills, while others appreciated the Health Advocate’s attention to identifying community-based resources for specific social risks. Specific tasks that participants reported feeling supported with included: filling out paperwork for community-based resources, finding ways to reduce non-transplant expenses (eg, utility bills, gas), establishing primary care, and establishing individual educational plans at school.
3. *We were proactive and accessible, regardless of the complexity of the support needed.* Participants broadly appreciated the Health Advocate’s ability to provide more intensive support than that which was offered by other members of the transplant team. Multiple participants discussed how the Health Advocate was able to respond more promptly and provide more exhaustive support than they were previously used to. They also cited that the Health Advocate was proactive in asking about their needs, rather than waiting for the participants to bring up new needs.

“A social worker, maybe because they have so many parents to deal with, focuses only on the patient’s illness. [Health Advocate] looks beyond that—at the parents and at problems that they normally don’t ask about or help you find resources for.”—ID 40, 30-day check-in

Participants cited that the support provided by the Health Advocate contributed to decreasing care burdens and, in turn, to reducing caregiver stress levels. These changes enabled them to be better able to care for their child. “I do feel less stressed, and I do get to not be on my phone a lot, where I can just put my phone down and just have even my alone time or just be with my kids.”—ID 26, 60 days (Table 2).

**TABLE 2:** Supporting quotes from qualitative interviews

Theme	Quote	Participant ID	Interview time point
Communication with the Healthcare team	Sometimes I had questions for the doctors, and [Health Advocate] made it easier for me to communicate with them.	ID 40	90 days
	[Health Advocate] can tell [clinical team], “she is going through this” or “she has this concern,” and I know [Health Advocate] will tell her.	ID 4	90 days
	I feel [clinical team] is paying more attention to me, paying more attention to the things I ask them for.	ID 29	30 days
	She also helps me clear up any doubts I have about medication, and she always asks if there’s anything she can help me with. She speaks with the doctors, and then the doctors contact me. The last time we spoke, my phone service was cut off, and she told me there were programs I could apply for to get a discount, so those are financial assistance programs that help me.	ID 40	30 days
	She’s being seen more quickly for everything.	ID 25	60 days
Multiple disparate tasks	Whether it was for appointments, medication refills, or questions about some results. She also helped me find immigration resources. She assisted with programs for rent assistance, which has been a struggle for me. What else? She helped me a lot.	ID 40	90 days
	I feel like that advocate is more. I won’t say willing to help, it seems like has more avenues, has more ability to help.	ID 30	30 days
	They help you in every way, they help free your mind from so much stress, and relieve so much frustration by helping you so much. They help parents a lot because you suffer from the trauma of having a sick child, and then you have to stress about getting paperwork, making medical appointments, and getting help if you need it. [Health Advocate] even helped me reduce my electricity bill. I used to pay 450, and now I pay less.	ID 29	90 days
	[Hepatologist] told me that you were able to speak with MOP and spent 2 hours on the phone with CalFresh! Thank you so much for doing that, I just looked back at my note and realized that I totally forgot to e-mail that information to mom, so I really appreciate you following up!	Social worker	Email
	She tells me about the available programs I am not aware of; where they give out food, and those things. She tells me about the assistance that may be available and has also helped me with it. Before they gave me [patient] insurance, I didn’t understand why they took it away. She explained to me why.	ID 4	30 days
	I asked for her permission to establish care with a primary pediatric office, book her a vision and dentist appointment. Edith gave me the okay. I spent 30 minutes on the phone with United Health Center of Mendota, California, booking all three appointments. Since [patient] is experiencing headaches, they were able to set the appointment for her next week.	ID 25	Health Advocate journal
	She always helped me to get the medicines right away. Because they sometimes take too long to give you the medications.	ID 28	90 days
	She intervened to have them put a little more effort into her, to give her a special class in school. She was the one insisting on the school, she was helping me with that. When the conference came, we did a video call, she also spoke with the teacher about some things, and about someone who was supposed to be there at the conference but wasn’t.	ID 25	90 days
Reduce stress/burden	She even helped relieve some of my stress with the assistance and answers she provided.	ID 40	90 days
	It’s just that in my mind, I don’t stress as much anymore.	ID 29	30 days
	Honestly, I feel less stressed [about finances].	ID 40	60 days
	I do feel less stressed, and I do get to not be on my phone a lot, where I can just put my phone down and just have even my alone time or just be with my kids.	ID 26	60 days
Availability	The social worker is very busy.	ID 40	60 days
	When I have a question, I send her a message and she responds quickly.	ID 29	60 days
	With the Health Advocate, she’s really on top of stuff and she’s always following up and she’s always asking if I need anything. When it comes to social worker, social workers are a little bit harder to get ahold of.	ID 26	60 days
	A social worker, maybe because they have so many parents to deal with, focuses only on the patient’s illness. [Health Advocate] looks beyond that—at the parents and at problems that they normally don’t ask about or help you find resources for.	ID 40	30 days
	The Health Advocate not only helps you with resources, but also asks how my son is doing, how he is doing at medical appointments, if we need help with medications, etc. A social worker focuses primarily on resources.	ID 13	90 days
	With the Health Advocate, it is different because they are the ones who call me. They ask me what I need, how my family is, what resources they can send me. A social worker is different. Yes, they are going to help me, but I have to tell them what I need.	ID 13	90 days
	The difference I see between the social worker and the Health Advocate is that the Health Advocate is always there, and the response is quick.	ID 36	30 days
	I like that she always answers when I call her whenever I need an answer or something I need.	ID 23	60 days
Acceptability	They help you in every way, they help free your mind from so much stress, and relieve so much frustration by helping you so much. For me, if this program stayed, I would be very happy.	ID 29	90 days
	[Health Advocate] took [patient]’s case very seriously, and I honestly have nothing bad, yes. It’s been a very positive journey with [Health Advocate].	ID 26	90 days
	They try to help people with things that are difficult for example	ID 23	30 days
	I enjoyed and liked having that support, as I mentioned. Because sometimes you feel alone and don’t know who to ask for advice.	ID 16	90 days
	It’s like Aladdin’s lamp; I touch it, and they come to my rescue.	ID 29	30 days
	I feel like I have someone I can trust.	ID 28	30 days

## DISCUSSION

We developed and pilot-tested a Health Advocate intervention tailored to the needs of families living post-liver transplant to address families’ experiences of social adversity. We observed improvements in *all quantitative* parent-reported and clinical outcomes study measures, suggesting that such an intervention could have health benefits. Qualitative data from caregivers supported the quantitative findings, revealing high levels of intervention acceptability. Moreover, we demonstrated that implementing the Health Advocate program and study procedures associated with program assessment was highly feasible. These results suggest that providing targeted supports using Health Advocates to address social adversity may be a viable strategy to improve pediatric liver transplant outcomes, laying the research groundwork for a larger, multi-center randomized clinical trial to test this intervention in the unique context of liver transplantation.

The qualitative feedback from this pilot was overwhelmingly positive. Many of the caregivers commented that they wished this program were available to them earlier in the transplant process. The demands on families over the first year of transplant are much higher than in later years—Health Advocates could be particularly helpful during this period, and this feedback should be incorporated into future work. These qualitative data suggest the program works by (1) addressing unmet social needs, (2) addressing health system complexity, and (3) providing emotional support to caregivers. This finding mirrors reports in the literature documenting that social care may improve health outcomes by strengthening emotional support and trust with the clinical team.^20^^–^^24^ The Health Advocate customized their intervention strategy to the needs of each individual participant. Some examples of the support provided included (1) finding and attending a legal aid phone call for a landlord dispute, (2) helping a parent format a resume and find a job, (3) locating neighborhood food banks, (4) finding free immigration services, and (5) attending a meeting with a child’s school to provide context for the Individual Education Plan (IEP). While much of the support provided did not require advanced training, we estimate the Health Advocates spent an average of 3.8 hours per participant in needs assessment, in addition to gathering and providing information about support services.

Despite the intensive nature of the intervention, this strategy could be a cost-effective solution to addressing disparities in the transplant system. In this pilot, the Health Advocate provided several supports that could yield large cost savings to both health systems and payors by facilitating timely access to healthcare. For example, the added support in scheduling a ride to a clinic appointment for families experiencing transportation challenges could reduce no-show rates, improve clinic efficiency, and result in cost savings to the health system. Similarly, helping families navigate challenges with immunosuppression refills could lead to improved adherence and reduced risk of immunosuppression-related complications (eg, T-cell-mediated rejection—TCMR), which would result in cost savings to health insurers. Our Health Advocate was a college graduate with no advanced medical training and with an entry-level job title. In contrast, social workers have advanced degrees with associated higher salaries, and our qualitative interviews with social workers uncovered that they often feel overburdened by their workloads.^9^^,^^10^ With the Health Advocate spending about 4 hours per participant, this model could offer a low-cost and scalable approach to providing social care support for families throughout the transplant process. Therefore, we expect the Health Advocate role to complement rather than replace or extend social work support. Future cost–benefit analyses to health systems and payors could identify strategies for funding this and similar interventions.

We previously found that children living with household social adversity experience increased risk of adverse outcomes.^7^ We co-produced this Health Advocate program specifically with and for families of children with recent liver transplants using rigorous human-centered design methods.^15^ In contrast to other navigation programs focused solely on healthcare navigation, the resulting program piloted in this study focused on helping families address social adversity (eg, food insecurity or housing instability) *in addition* to helping families manage challenges they faced navigating the healthcare system. Evidence for the addition of social care navigation (in addition to healthcare system navigation) suggests that it leads to improved health outcomes in primary care settings.^11^^–^^13^^,^^25^ However, evidence of similar programs focused on addressing social adversity in subspecialty pediatrics remains limited,^26^ but emerging data suggest they could improve clinical outcomes.^27^^,^^28^ Integrating social care into pediatric hepatology offers an opportunity to provide holistic care, in line with integrative hepatology.^29^^–^^31^

Given the wide variability in transplant team structures and resources, multi-center effectiveness and implementation data will be essential before recommending that the Health Advocates be scaled. Since starting this pilot, we have identified specific social risks (ie, food insecurity, housing instability, transportation challenges, and utility needs) at the time of transplant that predict later risk of T-cell-mediated rejection and increased healthcare utilization over the 2 years following liver transplantation.^7^^,^^32^ Future studies of intervention effectiveness should specifically target recruitment for these high-risk families to better understand this intervention’s ability to address disparities. Moreover, future studies should include outcomes important to a broad range of stakeholders. Here, we observed decreases in MLVI, an objective biomarker of immunosuppression adherence that predicts risk of TCMR,^33^ across the study period. Using the SIREN social care logical model as a guide,^20^ future studies could include clinical outcomes (eg, TCMR) and parent-reported outcomes (eg, caregiver burden) that are consistent with this theory of effectiveness. Moreover, in focus groups we held with our community advisory board to design future studies, parents identified child quality of life, burden of morbidity (eg, number of TCMR episodes), and markers of liver inflammation as priority outcomes they wish to see measured. These results underscore the importance of designing future studies specifically to assess the effectiveness of this intervention on priority outcomes.

This study should be interpreted in the context of the following limitations. First, this was a pilot study that was not powered to detect clinically meaningful differences in clinical outcomes. The intention was to measure the feasibility and acceptability of the study. We nonetheless observed improvements in all parent-reported and clinical outcomes. Second, we used broad enrollment criteria based on subjective assessments of who would “benefit” rather than selecting the highest risk patients. Our goal was to increase the generalizability of this intervention before widespread testing. Future studies could include high-risk groups, such as children with household material economic hardships, as this may optimize the power to detect improvements in clinical outcomes.^7^ Third, many caregivers reported favorable survey responses before the intervention, potentially creating a ceiling effect for the caregiver-reported outcomes. That we still observed improvements in these outcomes suggests that the intervention could be a viable strategy for improving parent/patient experience. Finally, social desirability bias in pre-post responses could limit interpretability. However, we took several steps to minimize this bias, including having a second CRC who was responsible for obtaining consent and measuring outcomes.

We found that this pilot intervention was feasible to implement and evaluate. Qualitative feedback from participants suggested strong intervention acceptability. Despite the small number of participants, we also saw positive impacts across all quantitative outcome measures. Well-powered studies should test the effectiveness of this intervention in reducing transplant-related complications.

## Supplementary Material
