# Supplementary material for: Health advocates to address social adversity in pediatric liver transplantation: A clinical trial
Source: Hepatol Commun. 2026 Jul 15;10(8):e00999. doi: 10.1097/HC9.0000000000000999 (PMC13671608; doi:10.1097/HC9.0000000000000999)
Supplement: Supplementary file 1 [file hc9-10-e00999-s001.docx]

Supplemental Figure 1

Reasons for drop out

N=2 Did not begin study activities

N=1 Lost to follow-up after 60 days of intervention

Reasons not consented

N=1 Lack of time

N=1 No current need for support

Total Completed Study

N=15 (83%)

Total Consented

N=18 (82%)

Total Approached

N= 22

Total Screened

N= 41
